# A Novel Capsule Lumbar Interbody Fusion (CLIF) in Treating Foot Drop due to Lumbar Degenerative Diseases: a Prospective, Observational Study

**DOI:** 10.1155/2021/6880956

**Published:** 2021-11-12

**Authors:** Kaiqiang Sun, Feng Lin, Jialin Jiang, Jingchuan Sun, Jiangang Shi

**Affiliations:** Department of Orthopedic Surgery, Changzheng Hospital, Naval Medical University, 415 Fengyang Road, Shanghai 200003, China

## Abstract

**Objective:**

This present study aimed to explore the clinical effects of a novel capsule lumbar interbody fusion (CLIF) on foot drop due to lumbar degenerative diseases.

**Methods:**

Between June 2018 and January 2019, a total of 27 patients admitted to our department with lumbar degenerative diseases with associated foot drop were prospectively enrolled. Given the selection of surgical technique, patients were divided into traditional TLIF group and CLIF group. We assessed patients' neurological status using JOA and VAS score, tibialis anterior muscle strength using MMT score, diameter and hemodynamic parameters of the L5 nerve root using intraoperative ultrasonography (IoUS), and related radiological parameters of the lumbar spine. Operation time, blood loss, and surgery-associated complications were also recorded.

**Results:**

The median duration of follow-up was 150 (6–1460) months. At the final follow-up, all patients acquired satisfactory improvement of neurological function. However, patients in the CLIF group showed better early recovery of foot drop three months after operation than those in the TLIF group, with 75% excellent rate. In addition, IoUS suggested that the diameter and hemodynamic parameters of the L5 nerve root were improved better in the CLIF group, which may suggest the correlation between the recovery of foot drop and the status of L5 nerve root. No severe complications were encountered with CLIF.

**Conclusions:**

Our preliminary study revealed that the axial tension of L5 nerve root may be involved in the pathological mechanism of foot drop. The novel technique of CLIF can shorten the lumbar spine and can be effective and safe for the treatment of foot drop due to lumbar degeneration-related diseases.

## 1. Introduction

Foot drop has typically been denoted as a condition with paralyzed or weak tibialis anterior (TA) muscles and even dysfunctional motor function. Patients with foot drop frequently experience stumble or even fall during walking [1]. In fact, foot drop resulting from spinal diseases is not rare in spine-related clinical practice [2]. However, among the massive spinal causes for foot drop, lumbar degenerative diseases (LDD) are the most common [3]. In addition, those patients frequently exhibit lumbar disc herniation (LDH) and lumbar spinal stenosis (LSS), with the L4/5 spinal level being the most affected [4].

LDD-mediated foot drop is an entity significantly different from that of peripheral neuropathy. Although previous studies have described the manifestations of foot drop and its clinical treatments, the clinical recovery of foot drop caused by LDD remains unsatisfactory [5]. Previous studies focused too much on the effects of factors such as duration of palsy and preoperative TA muscle strength on the recovery of foot drop [4–6]. However, disputation still exists. Our previous study found that impairment caused by axial traction of the lumbar nerve root may be another major contributor to symptoms including pain, numbness, and weakness of low extremities in patients with LDD and recommended that the decompression of the lumbar spine should not only include the management of the surrounding compression (herniated disc or narrow intervertebral foramina) of the neural elements but also include the release of the axial tension of the nerve root [7, 8]. However, whether the release of the lumbar nerve root is effective to the recovery of foot drop remains unknown.

Hence, in this present study, the technique of CLIF was designed to reduce of the axial tension of the neural elements in the lumbar spine, which essentially means rod compression before inserting the interbody fusion cage combined with spine shortening. This present study aimed to investigate the effects of CLIF on the recovery of foot drop caused by LDD.

## 2. Methods and Materials

### 2.1. Patients' Population

We conducted a single-centered, prospective, observational study with patients who had LDD, associated with foot drop, from June 2018 to January 2019 in the Spine Center of Changzheng Hospital, Shanghai, China. All the enrolled patients had complete medical records, including X-ray and magnetic resonance imaging (MRI).

All patients were indicated for surgery due to severe LSS with/without LDH. Patients would be excluded if they had concomitant other diseases causing foot drop such as tumor [9], trauma [2], disc herniation at cervical or thoracic spine [2, 10], or inflammation-related diseases such as multiple sclerosis [11]; if they had peripheral neuropathy (peroneal neuropathy) due to external compression, nerve entrapment, iatrogenic factors, weight loss, and diabetes [12, 13]; if they had previous spine surgery; or if they had incomplete medical data during the follow-up. In addition, considering the major contribution of L5 nerve to foot drop, all patients enrolled in this study had surgery levels including L4/5 level.

This study was performed in accordance with the principles of the Declaration of Helsinki and was also approved by the Ethics Committee of our hospital.

### 2.2. Selection of Surgical Technique

We designed a prospective, observational study enrolling patients with diagnosis of foot drop due to lumbar degenerative diseases. The study period was determined from June 2018 to January 2019. All the patients with foot drop resulting from LDD referred to our clinic during this period would be recruited, and a total of 27 patients were finally enrolled. All patients enrolled would be informed of the benefits and potential risks of these two techniques before operation and came to a consensus to participate in this study. Subsequently, patients would be divided into TLIF group and CLIF group based on the patients' acceptance and doctors' experience. The duration of follow-up lasts for at least 12 months.

### 2.3. Surgical Technique

**Figure 1:**
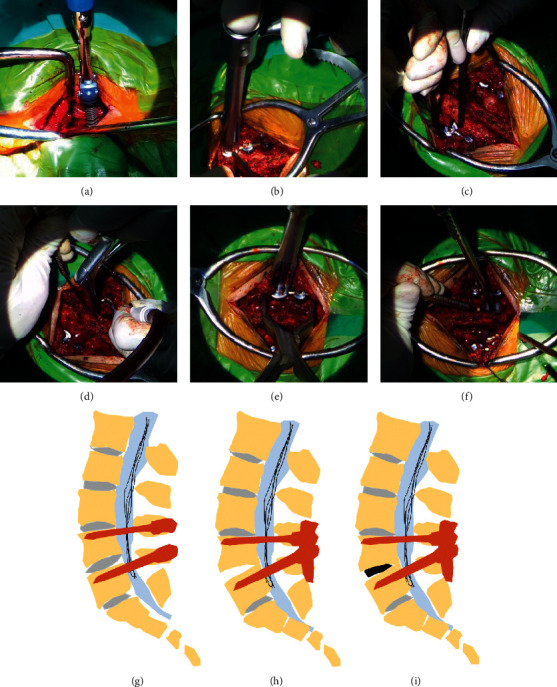
Representative intraoperative images of CLIF: (a) position of surgical segment; (b) insertion of ipsilateral pedicle screws and rods; (c) insertion of contralateral pedicle screws and rods; (d) removal of the disc tissue and partial cartilage endplate; (e) slow axial compression of the operated segment; (f) insertion of the intervertebral cage; (g) insertion of the pedicle screws; (h) installation of the rods prior to the placement of cages; and (i) axial compression of the segment after insertion of the cage. CLIF: transforaminal lumbar interbody fusion with spine shortening.

The TLIF surgery has been described in detail in previous studies [14, 15]. Here, we presented a case who required lumbar surgery at L4/5 level to illustrate the procedure of CLIF. Briefly, under general endotracheal anesthesia, the patient was placed in a prone position. Firstly, the surgical segments would be confirmed (Figure 1(a)). Subsequently, the pedicle screws were inserted bilaterally in L4-L5 segments. Intraoperative fluoroscopy was used to confirm the good position of screws. Next, the interspinous ligaments between L4 and L5 were resected, with the preservation of the spinous process for later spine compression. The necessary facetectomy on the symptomatic side was performed to achieve adequate decompression of the stenosis, and the superior articular process in the lower vertebra and the inferior articular process in the upper vertebra on both sides were resected with the pedicle preserved to achieve the decompression of the ipsilateral dural sac and nerve roots and intraoperative ultrasound examination. In addition, the contralateral facet joint was managed according to this procedure. Then, the ligamentum flavum was removed bilaterally.

However, different from traditional TLIF, the fixation and tightening of the rods (Figures 1(b) and 1(c)) were performed prior to the placement of the cage followed by necessary discectomy and removal of the cartilage endplate (Figure 1(d)). Notably, slow compression to the operated segment was also performed prior to the insertion of the cage but after the fixation of the rods (Figure 1(e)). Then, a nerve probe was used to evaluate the tension of the nerve root followed by the insertion of the cage (Figure 1(f)). With regard to cage size, a test module would be used prior to cage being implanted into the L4/5 intervertebral space. The surgeon must make sure that the spinous process gap and intervertebral space were appropriately shortened, and the nerve root was loosened. The whole concept of CLIF is illustrated in Figure 1 (Figures 1(g)–1(i)). An intraoperative ultrasound was used to evaluate the condition of the nerve root before and after spine shortening. Patients were suggested to wear a waist support for 12 weeks after surgery.

### 2.4. Clinical and Radiological Examination

The neurological function of patients was assessed using the Japanese Orthopaedic Association (JOA) score, and the pain symptoms were assessed by the visual analogue scale (VAS score). Other parameters including operation duration time, intraoperative blood loss, and surgery-associated complications were also recorded.

**Figure 2:**
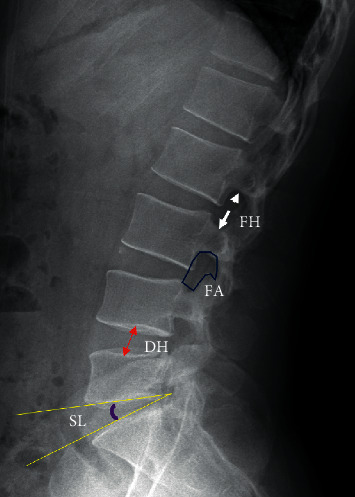
Illustration of the radiological measurement based on X-rays. FH: foraminal height; DH: disc height; FA: foraminal area; SL: segmental lordosis.

All patients accepted X-rays, MRI, and/or CT before operation. Considering the surgical levels of all patients with foot drop in this study involved L4/5, we chose the height of intervertebral space (HIS), foraminal height (FH), foraminal area (FA), and segmental lordosis (SL) at the level as the research parameters (Figure 2) [8].

The HIS was defined as the distance between the midpoints of cephalic and caudal endplate of the intervertebral space, which was used to evaluate the effect of spine shortening [16]. FH denoted the maximum distance between the lower margin of the superior pedicle and upper margin of the inferior pedicle [8]. FA was determined as illustrated in Figure 2. These two parameters were used to evaluate the potential compression of spine shortening on the nerve root at intervertebral foramina. SL was defined as the angle between the lines parallel to the inferior endplate and the superior endplate of the index disc, which was used to assess the lumbar alignment [8].

### 2.5. Evaluation of Nerve Root Using Intraoperative Ultrasonography (IoUS)

IoUS has been previously used in spine surgery, including disc herniations, spinal stenosis, and pedicle screw instrumentation, due to its clear definition of normal structures and pathologic lesions [17, 18]. As reported previously, when the nerve root was pulled axially, the diameter and blood flow volume would decrease [19]. Therefore, IoUS was firstly used to evaluate the effect of CLIF with spine shortening on the axial tension of neural elements via changes of the diameter and blood flow volume of the L5 nerve root in this present study.

**Figure 3:**
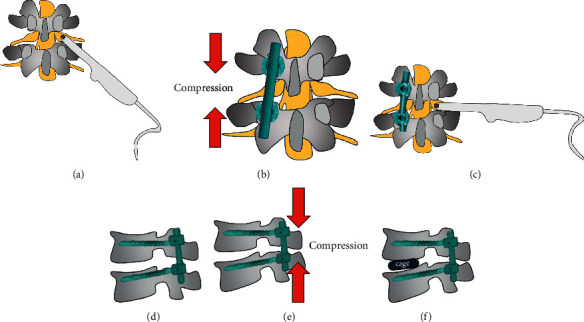
Illustration of the utility of IoUS during operation: (a) measurement of the condition of L5 nerve root before spine shortening; (b) slow spine compression; (c) measurement of the condition of L5 nerve root after spine shortening; (d) sagittal view of the affected segment before compression; (e) sagittal view of slow spine compression; and (f) sagittal view of cage insertion.

Figure 3 shows the details of IoUS measurement before and after shortening. Briefly, IoUS was performed using a water-path imaging technique to investigate the hemodynamic parameters of the L5 nerve root using a digital echo camera (APL10 300 TUS-A300, Prosound a10; TOSHIBA Medical Co., Tokyo, Japan) after resection of the spinous process, lamina, and ligamentum flavum and a 3.5–11 MHz linear array transducer before spine shortening (Figure 3(a)). The ultrasound transducer was directed perpendicular to the horizontal plane as much as possible to obtain an accurate axial section of the exiting part of L5 nerve root and was stabilized for several seconds to prevent motion blur in the video (Figure 3(a)). After spine shortening, the dynamics of the L5 nerve root was measured again (Figures 3(b) and 3(c)), and the hemodynamic parameters of the L5 nerve root would be acquired before and after spine shortening. The sagittal view is shown in Figure 3 (Figures 3(d)–3(f)). In addition, to obtain the blood volume of L5 nerve root, a radiocontrast agent (sulfur hexafluoride) was used. After injection of sulfur hexafluoride, the timer would be initiated. Subsequently, the time duration and the slope ratio of time curve before the radiocontrast agent reached the peak, as well as the value of the peak would be recorded before and after spine shortening, respectively. The curve indicated the concentration changes of the radiocontrast agent over time, and the faster the curve increased, the more the blood flow volume.

### 2.6. Diagnosis and Evaluation of Foot Drop

The foot drop was diagnosed mainly based on the tibialis anterior (TA) strength and medical history, and the manual muscle test (MMT) was utilized to assess the muscle strength of TA [2]. In this study, foot drop would be diagnosed when muscle strength of TA was below or equal to 3 (out of 5) [2]. The symptoms duration of foot drop was defined as a period from onset of stumbling or weakness of ankle dorsiflexion to surgery. The previous study has indicated that the optimal time for improving foot drop after surgical intervention was 6 weeks, and thus in this study, we evaluated the patients' recovery at preoperation, six weeks after operation, and one year after operation [19].

The recovery grade of foot drop ranged from “excellent” to “poor” based on the postoperative MMT score of TA [2]. Excellent denotes that the MMT score was grade 4 or 5; good denotes grade 3; fair denotes improvement but still below 3; and poor indicates no any recovery until the last follow-up [2]. We also evaluated the recovery rate of TA muscle strength: (grade at the last follow-up − grade before operation)/(5 − grade before operation) × 100% [2]. Grade 3 was considered grade 2.5 in this study.

### 2.7. Statistics Analysis

Statistical analysis was carried out using GraphPad Prism 9 (GraphPad Software Inc, La Jolla, CA). Data in this present study were presented as the median value. The Mann–Whitney *U* test was used to detect the statistical differences of demographic parameters (patients' age, symptoms duration, intraoperative blood loss, and operation time), radiological outcomes (HIS, FH, FA, and SL), IoUS parameters, and clinical scores (JOA score and VAS score) between the two groups, and intragroup comparison was conducted via the Wilcoxon signed rank test. Fisher's exact test was used to compare the gender, clinical tests, surgical segments, and comorbidities between the two groups. To further explore the relationship between foot drop and L5 nerve root, we performed the correlation analysis. Values that were less than 0.05 (*p* < 0.05) were considered statistical significance.

## 3. Results

There were 15 patients in the TLIF group (3 females and 12 males) and 12 in the CLIF group (3 females and 9 males) (*p* > 0.999). The median age of patients in the TLIF group was 43 (27–60) years, not statistically different from those in the CLIF group, 46 (26–69) years (*p*=0.895). There were no statistical differences between these two groups with regard to duration of symptoms/foot drop, surgical segments, intraoperative blood loss, and operation time (all *p* > 0.05) (Table 1).

Table 2 shows the clinical scores of patients in the TLIF group and CLIF group. No significant differences were observed regarding patients scores, including JOA score and VAS score at preoperation, three months after operation, and the final follow-up (all *p* > 0.05). At the final follow-up, all patients in this present study acquired satisfactory recovery regarding VAS and JOA scores (both *p* < 0.05). In terms of the MMT score, all patients acquired improvement after operation. However, patients in the CLIF group exhibited better early recovery of foot drop, as indicated by the MMT score and its recovery rate at three months after operation (*p*=0.025). At the final follow-up of one year, almost all patients reported satisfactory recovery of MMT score, without statistical difference between the two groups (*p*=0.065). No surgery-related complications were observed perioperatively.

In order to investigate the effects of spine shortening, we firstly analyzed the radiological changes at surgical segment. Considering the major contribution of L5 nerve to foot drop, the surgical levels at L4/5 were chosen. We evaluated the changes of HIS, FH, FA, and SL at this level. As shown in Table 3, the HIS of L4/5 for patients in the CLIF group was decreased in comparison with preoperation and that of patients in the TLIF group postoperatively (both *p* < 0.05), which indicated the surgical segment was shortened after operation, and we believed this was the major reason for the satisfactory recovery of foot drop in this present study. We further evaluated the changes of morphology of intervertebral foramina at L4/5 and found that the FH and FA in the CLIF group were slightly lower compared with the TLIF group but without statistical differences (both *p* > 0.05). In fact, during CLIF or TLIF, the bilateral intervertebral foramina decompression was frequently carried out in order to avoid the stenosed foramina after operation, which may result in this result. In addition, no statistical difference was detected regarding SL between the two groups before (*p* > 0.999) and after operation (*p*=0.952).

In addition, we evaluated the condition of the L5 nerve root using IoUS. As shown in Table 4, the median diameter of L5 nerve root after decompression in two groups was both improved compared with preoperation (both *p* < 0.05). However, patients in the CLIF group had a higher increase of diameter than those in the TLIF group (*p* < 0.05). We further analyzed blood flow volume of the focal L5 nerve root at surgical level and found that the postoperative time interval before peaking in the CLIF group was significantly shorter than that of the TLIF group (20.8 vs. 27.8) (*p*=0.019). In addition, the postoperative peak value of L5 nerve root in the TILIF-SS group was higher than that in the TLIF group (4.8 × 10^−5^ vs. 3.7 × 10^−5^) (*p*=0.002).

**Figure 4:**
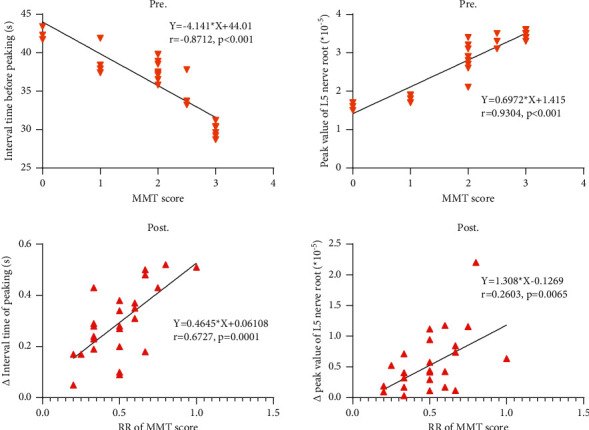
Correlations of the MMT score and IoUS parameters (a) before and (b) after operation.

Correlation analysis showed that both the preoperative time interval before peaking (*r* = −0.8712, *p* < 0.001) and the peak value (*r* = 0.9304, *p* < 0.001) were negatively and positively related with the preoperative MMT score, respectively, which indicated the potential contribution of L5 nerve root injury to foot drop (Figures 4(a) and 4(b)). In addition, the recovery rate of MMT score at three months after operation also correlated positively with the changes of time interval before peaking (*r* = 6727, *p*=0.0001) and the peak value (*r* = 0.2603, *p*=0.0065) (Figures 4(c) and 4(d)).

### 3.1. Case Presentation

**Figure 5:**
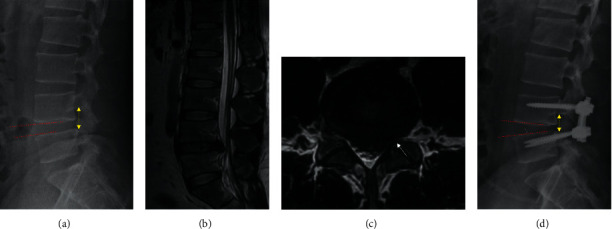
Images of the case: (a) preoperative lateral X-rays; (b) preoperative sagittal MRI; (c) preoperative axial MRI; and (d) postoperative lateral X-rays.

A 34-year-old female patient with numbness and pain at her left lower extremity and foot drop for nearly four months was admitted to our institution. Physical examination showed she had positive Lasegue sign of 45°. The MMT score of her left tibialis anterior (TA) was 3 and 5 in her right. Preoperative images indicated that there was a herniated disc compressing neural elements at her L4/5 segment, with the loss of lumbar lordosis (Figures 5(a)–5(c)). A single-level CLIF at L4/5 was given (Figure 5(d)). Six months after operation, the patient had significant improvement regarding neurological function with better lumbar lordosis. More importantly, her dropped foot had satisfactory recovery, with the MMT score of 5.

**Figure 6:**
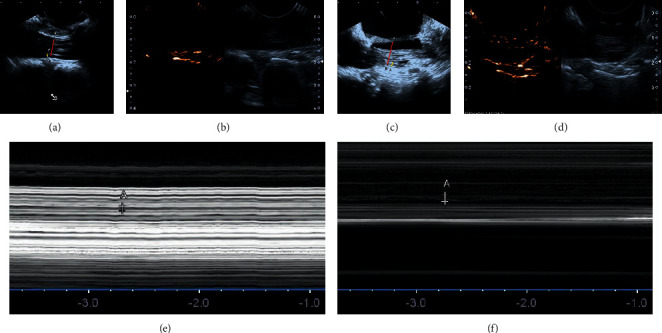
Intraoperative ultrasonography (IoUS) of the case: (a) preoperative diameter of the L5 nerve root (yellow line) and dura mater (red line); (b) preoperative blood perfusion of the L5 nerve root (white arrow); (c) postoperative diameter of the L5 nerve root (yellow line) and dura mater (red line); (d) postoperative blood perfusion of the L5 nerve root (white arrow); (e) preoperative amplitude of the L5 nerve root; and (f) postoperative amplitude of the L5 nerve root.

Figure 4 shows the IoUS of the patients before and after spine shortening. Before we compressed the lumber spine, the diameter of the nerve root and dura mater was 1.5 mm and 10.1 mm, respectively (Figures 6(a) and 6(b)). The blood perfusion of the L5 nerve root was also improved significantly compared with the poor perfusion before spine shortening (Figures 6(c) and 6(d), white arrow). In addition, the amplitude of L5 nerve root was also increased from 1.1 mm before spine shortening to 1.6 mm after shortening (Figures 6(e) and 6(f)). These improved IoUS parameters suggested the patient's nerve root tension acquired satisfactory axial release via spine shortening.

## 4. Discussion

Foot drop resulting from LDD has been a hot topic of interest among spine surgeons. Although previous studies have been investigating the mechanism of LDD-derived foot drop and related treatments, the results are still disputed. Anatomically, the TA muscle and extensor hallucis longus were mainly innervated by the L5 nerve root [5]. Aono et al. reported that an impairment at the L5 nerve root was the main contributor to foot drop [1]. In this present study, to minimize the effects of other potential risk factors, all patients enrolled presented with lesion involving the L4/5 level. In addition, to evaluate the changes of the L5 nerve root on the recovery of foot drop, we firstly introduced IoUS during operation. Interestingly, the results of this present study suggested that the severity of foot drop before operation correlated with the blood volume of L5 nerve root. Additionally, the recovery of foot drop was also associated with the changes of the blood volume of L5 nerve root. Therefore, we in this present study confirmed the vital role of the function of L5 nerve root in foot drop. However, McCulloch and Waddell found that except the L5 nerve root, L4 and S1 nerves also innervated certain part of TA based on electrical stimulation [20]. A study by Iizuka et al. also showed that L4 or S1 was also affected in most of the patients with LDH-induced foot drop, and they attributed it to a transitional vertebra, namely lumbarized S1 or sacralized L5 [5]. McCulloch and Waddell ever suggested that the functional L5 root came from the most caudal lumbosacral segments [20]. Therefore, we deduced that the function of S1 root may become more like that of L5 root when there is lumbarization of S1, and that the function of L4 root may become more like that of L5 root when there is sacralization of L5. However, it was reported that a lesion in the thoracolumbar spine (T11-L2) could also cause foot drop, and that foot drop lesion was located at the epiconus [2]. Therefore, foot drop may be a multifactorial disease, and our present study only showed the role of L5 nerve root in foot drop. Further study is needed to explore the exact mechanism of LDD-derived foot drop.

Previous studies have investigated the clinical outcomes of patients with foot drop. However, patients did not acquire satisfactory recovery in spite of sufficient decompression. Eysel et al. performed a study of 240 patients with LDH and found that the postoperative recovery rate was only 40% for patients with grade 2 paresis [21]. Aono et al. reported that only 61% patients had various degrees of functional recovery after lumbar operation, and that 28% had no improvement [1]. Ghahreman et al. reported that only 41% patients who underwent surgical decompression acquired full recovery, with 21% unchanged [22]. A study of Iizuka et al. showed patients with LDH acquired better recovery than those with LSS, and the overall recovery rate was 40% [5]. However, previous studies mainly focused on the routine surgical decompression [1, 5, 21, 22]. Our previous study found another ignored impairment caused by axial traction of the lumbar nerve root may be another major contributor to lumbar symptoms in patients with LDD [8]. Hence, in this present study, the technique of CLIF was designed to reduce the axial tension of the nerve root in the lumbar spine and the surgical outcomes of CLIF were comparable with those of TLIF. As shown in the results, despite the improvement of foot drop in all patients after operation, patients in CLIF acquired better early recovery compared with those in the TLIF group, with 75% patients had excellent recovery at three months after operation. Based on the results above, we deduced that TLIF with spine shortening may facilitate to early recovery of foot drop. In fact, early recovery frequently affects patients' final recovery. As reported by Ghahreman et al., the most significant improvement of ankle weakness occurs within the first 6 weeks, without substantial improvement after that [21]. Therefore, promoting early recovery of foot drop is significantly important to patients' long-term prognosis. Notably, no statistical difference was observed regarding the MMT score at the final follow-up between the two groups, whereas patients in the CLIF group seemed to have higher MMT scores, which we deduced may correlate with the small sample in this present study.

To further confirm the favorable recovery of foot drop in the CLIF group, we focused on the effect of surgical techniques on the L5 nerve root. The diameter and blood flow volume of the L5 nerve root were evaluated. As shown in this study, patients in the CLIF group had a more increased diameter of L5 nerve root, compared with those in the TLIF group. In terms of blood flow volume of L5 nerve root, the time interval before peaking decreased more significantly in the CLIF group than the TLIF group. In addition, the peak value of blood flow volume in the CLIF group was also higher than that in the TLIF group. Collectively, the technique of CLIF resulted in better function recovery of the L5 nerve root. Furthermore, we also evaluated the changes of radiological parameters between the two groups and found that the HIS in the CLIF group was decreased. However, the procedure of spine shortening did not obviously stenosed the intervertebral foramina, as indicated by FA and FH. In fact, during operation, the bilateral decompression was frequently carried out unconsciously to avoid the stenosed foramina after operation. Therefore, the technique of CLIF was feasible and safe.

The prognostic factors for the recovery of foot drop due to LDD have been reported in several studies, with most risk factors being symptoms duration and preoperative TA muscle strength [22, 23]. In this present study, due to the limitation of sample, we did not make further risk analysis. However, patients with worse preoperative muscle strength of TA and longer symptoms duration had relatively bad recovery, consistent with previous studies [23, 24]. Taken together, timely treatment facilitates better recovery. However, it is notable that foot drop is frequently considered a sign for symptom severity of underlying LDD in clinical practice, and almost all the published cases received operation. Therefore, sound and comprehensive evidence in the selection of surgical or conservative treatments for foot drop are imperatively required. However, a RCT study demonstrated the absence of superiority of surgery over conservative therapy in treating LDD-derived foot drop [25]. Resultantly, the selection of surgery should include comprehensive evaluation of the LDD and not solely foot drop. Other clinical examinations, such as neurogenic claudication, might be also required in selection of conservative of surgical treatment.

Based on the results above, we deduced that the spine shortening led to decreased axial tension of the L5 nerve root and added the decompressive effect from an axial aspect to traditional decompression via only elimination of the compressed lesion, which may be the reason of the more satisfactory recovery of foot drop in the CLIF group. However, IoUS parameters were still indirect indicators to evaluate the tension of nerve root; the direct relationship between axial hypertension of nerve root and foot drop remains to be studied. An auxiliary instrument which can directly quantify the axial tension is required.

However, several limitations should be acknowledged here. First, this present study used both radiological and intraoperative ultrasonography results in order to reveal the spine shortening effects of CLIF. In addition, IoUS was firstly used to evaluate the condition of nerve root in this present study, which was a preliminary attempt. Therefore, we did not overstate the cases in regard to the ultrasonic findings at this time. However, our future study will further focus on the relationship between intraoperative ultrasonography and the function of nerve root during spine surgery and validate the effectiveness of this method, which may extend the application of intraoperative ultrasonography. Second, foot drop is a multifactorial disease, and there may be other types of stretching of the traversing L5 nerves, rather than axial stretching only. In fact, this preliminary study was designed based on the concept that axial stretching of the L5 nerve root may be another pathogenic factor to evaluate the effect of axial decompression of nerve root on the recovery of foot drop, and the encouraging results indicated that CLIF can be effective and safe for the treatment of foot drop due to lumbar degenerative diseases. However, our present study mainly focused on the axial decompression, and more studies are still required to explore the exact pathogenesis of foot drop. Third, although we conducted this study prospectively, this present study was inconsistent with the requirements of the RCT trial, and high-quality studies, such as RCT trial, will be carried out in the future to validate the outcomes of this study.

## 5. Conclusion

Axial hypertension of L5 nerve root may be involved in the pathological mechanism of foot drop, and transforaminal lumbar interbody fusion with spine shortening (TLIF-SS) can be effective and safe for the treatment of foot drop due to lumbar degenerative diseases. However, further studies with more cases will be required to validate its generalizability and safety.

## Figures and Tables

**Table 1:** Clinical characteristics of patients in the TLIF group and CLIF group.

Parameters	Total	TLIF	CLIF	*p*
Age (years, median (range))	46 (26–69)	43 (27–60)	46 (26–69)	0.895
Gender (*N*, female/male)	6/21	3/12	3/9	>0.999
Duration of symptoms (months, median (range))	12 (0.3–120)	12 (0.3–120)	10 (0.3–120)	0.837
Duration of foot drop (days, median (range))	150 (6–1460)	182 (6–365)	105 (7–1460)	0.761

Intraoperative parameters				
Operation time, (mins, median (range))	120 (75–260)	120 (75–230)	155 (100–260)	0.390
Blood loss (ml, median (range))	200 (50–1000)	100 (50–1000)	200 (50–600)	0.397

Surgical segments				
1 level	16	9	7	
2 levels	9	5	4	
4 levels	2	1	1	
Duration of follow-up (months, median (range))	19 (13–28)	19 (13–27)	19.5 (14–28)	0.513

**Table 2:** Clinical evaluation of patients in the TLIF group and CLIF group.

Parameters (median (range))	Total	TLIF	CLIF	*p* value
Pre				
VAS	3 (0–5)	3 (0–5)	3 (0–5)	0.511
JOA	16 (15–21)	16 (15–21)	15.5 (15–18)	0.211

3 months after operation				
VAS	1 (0–3)^*∗*^	1 (0–3)^*∗*^	1.5 (0–3)	0.799
JOA	20 (18–23)^*∗*^	20 (18–23)^*∗*^	20 (18–21)^*∗*^	0.530

Final follow-up				
VAS	0 (0–1)^*∗*^	0 (0–1)^*∗*^	0 (0–2)^*∗*^	0.281
JOA	24 (20–26)^*∗*^	25 (20–26)^*∗*^	24 (22–26)^*∗*^	0.448

Muscle strength of TA				
Pre. muscle	2 (0–3)	2 (0–3)	2 (0–3)	0.989
3 months after operation	4 (1–5)^*∗*^	3 (1–4)^*∗*^	4 (3–5)^*∗*^	0.025
Recovery rate (%)	50 (20–75)	33 (20–60)	55 (33–100)	0.008
Final follow-up	5 (3–5)^*∗*^	4 (3–5)^*∗*^	5 (4–5)^*∗*^	0.065
Recovery rate (%)	100 (50–100)	75 (50–100)	100 (50–100)	0.058

^*∗*^indicates a statistical difference of the parameter at different time points after surgery compared with that at preoperation. JOA: Japanese Orthopaedic Association; VAS: visual analogue scale.

**Table 3:** Radiological results of patients in the TLIF group and CLIF group.

Parameters (median (range))		TLIF	CLIF	*p* value
HIS (mm)	Pre	9.3 (7.6–10.3)	9.5 (8.9–11.5)	0.315
	Post	9.8 (8.6–10.8)	7.4 (6.6–9.2)	<0.001
	Change	1.0 (0.3–1.7)	1.9 (1.4–2.3)	0.249

FH (mm)	Pre	18.6 (11.6–23.7)	19.8 (13.5–22.9)	0.508
	Post	19.8 (12.5–24.6)	17.7 (12.4–19.4)	0.051
	Change	0.9 (0.2–1.2)	2 (1.1–3.5)	0.585

FA (mm^2^)	Pre	149.4 (134.2–160.2)	150.1 (139.2–161.4)	0.581
	Post	190.6 (179.5–205.7)	187.1 (179.1–198.2)	0.057
	Change	43.4 (32.0–55.8)	37.2 (27.9–47.8)	0.004

SL (°)	Pre	7.5 (0.9–9.3)	7.1 (1.2–9.1)	>0.999
	Post	7.8 (2.5–10.3)	8.0 (2.4–9.4)	0.952
	Change	0.7 (0.1–1.6)	0.6 (0.08–1.2)	0.523

HIS: height of intervertebral space; FH: foraminal height; FA: foraminal area; SL: segmental lordosis.

**Table 4:** IoUS parameters of L5 nerve root before and after surgery in the TLIF group and CLIF group.

Parameters (median (range))		TLIF	CLIF	*p* value
Diameter of L5 nerve root, mm	Pre	1.5 (1.3–1.6)	1.4 (1.3–1.6)	0.933
	Post	1.6 (1.5–2.0)^*∗*^	2.0 (1.9–2.2)^*∗*^	<0.001
	Change	0.2 (0.1–0.7))	0.6 (0.5–0.7)	<0.001

Time interval before peaking (s)	Pre	37.1 (30.3–41.9)	37.5 (28.7–43.4)	>0.999
	Post	27.8 (20.9–34.8)^*∗*^	20.8 (14.9–30.2)^*∗*^	0.019
	Change	8.6 (2.6–16.1)	12.7 (7.3–21.9)	0.038

Peak value of L5 nerve root (×10^−5^)	Pre	3.1 (1.6–3.5)	2.7 (1.5–3.6)	0.492
	Post	3.7 (1.9–4.7)^*∗*^	4.8 (3.5–5.9)^*∗*^	0.002
	Change	0.5 (0.1–1.9)	2.0 (1.0–3.3)	<0.001

^*∗*^indicates a statistical difference of the parameter at different time points after surgery compared with that at preoperation. IoUS: intraoperative ultrasonography.

## Data Availability

Data will be available when required.
